# The Art of Early Rising

**Published:** 1885-02

**Authors:** 


### THE ART OF EARLY RISING.

The proper time to rise is when sleep ends. Dozing should not be allowed. True sleep is the aggregate of sleep, or is a state consisting in the sleep or rest of all the several parts of the organism. Sometimes one and at another time another part of the body, as a whole, may be the least fatigued, and so the first to wake, or the most exhausted, and therefore the most difficut to arouse. The secret of good sleep is, the physiological conditions of rest being established, to work and weary the several parts of the organism as to give them a proportionally equal need of rest at the same moment; and, to wake early and feel ready to rise, a fair and equal start of the sleepers should be secured; and the wise self-manager should not allow a drowsy feeling of the consciousness or weary senses, or an exhausted muscular system, to beguile him into the folly of going to sleep again when once he has been aroused. After a very few days of self-discipline, the man who resolves not to doze, that is, not to allow some sleepy part of his body to keep him in bed after his brain has once awakened, will find himself, without knowing why, an early riser.—*The Lancet.*

